# Cell clone selection—impact of operation modes and medium exchange strategies on clone ranking

**DOI:** 10.3389/fbioe.2024.1479633

**Published:** 2025-01-20

**Authors:** Marie Dorn, Christine Ferng, Kerensa Klottrup-Rees, Kenneth Lee, Martina Micheletti

**Affiliations:** ^1^ Advanced Centre for Biochemical Engineering, Department of Biochemical Engineering, University College London, London, United Kingdom; ^2^ BioProcess Technologies and Engineering, Biopharmaceutical Developments, AstraZeneca, Gaithersburg, MD, United States; ^3^ Cell Culture and Fermentation Sciences, Biopharmaceutical Development, AstraZeneca, Cambridge, United Kingdom

**Keywords:** perfusion, fed-batch, small-scale, microwell plate, high-throughput, cell clone screening, Chinese Hamster Ovary cells, therapeutic antibody production

## Abstract

Bioprocessing has been transitioning from batch to continuous processes. As a result, a considerable amount of resource was dedicated to optimising strategies for continuous production. However, the focus has been on developing a suitable and scalable perfusion strategy with little attention given to the selection of optimal cell clones. Cell line development and lead clone selection are critical to bioprocess development. The screening and selection process is typically performed in stages. Microwell plates (MWP) are used to narrow down the number of clone candidates, which will undergo further selective screening in progressively larger small-scale bioreactors (12 mL–3 L) to identify the top clone for GMP production. Perfusion mode is typically applied at bench-scale for optimisation purposes, while process development and cell clone screening studies at mL-scale still commonly use fed-batch methods. The change of operation mode from bolus feeding to perfusion with a regular exchange of medium, leads to questions regarding the reliability and fit of initial clone selection. Is the early-stage clone ranking impacted by the discrepancy in the operation mode, and does this potentially result in the exclusion of cell clones suitable for perfusion processes? To address this question, we evaluated various CHO cell clones expressing two antibody products using MWP methodologies in fed-batch and semi-perfusion mode. We assessed growth, metabolic, and productivity performance, and ranked cell clones using two different strategies. The first strategy evaluated clones based on a single parameter: the cell-specific productivity (q_P_). The second considered a collection of multiple parameters using the metric of the Manufacturability index (MI_CL_). Both ranking strategies showed an impact of operation mode and perfusion rate on the clone ranking. Notably, depending on the chosen operation mode, different sets of candidate clones might have been selected for further, more extensive screening. Additionally, we evaluated the reproducibility of our results demonstrating consistency in cell clone growth performance and ranking.

## 1 Introduction

Since 2015, the biopharmaceutical industry has experienced significant growth, with 50–70 new product approvals per year (by tradename) for the combined US and European markets alone (Walsh and Walsh, 2022). This represents a 3- to 4-fold increase compared to the previous decade (2005–2014; Walsh, 2018; Walsh and Walsh, 2022). While new modalities such as antibody-drug conjugates and cell and gene therapies are increasingly being developed, traditional modalities such as monoclonal antibodies (mAbs) continue to represent a substantial proportion of all newly approved products accounting for approximately 53% (Senior, 2023; Walsh, 2018; Walsh and Walsh, 2022).

The pharmaceutical industry is experiencing a growing demand for more flexible, cost-effective, and time-efficient manufacturing platforms for therapeutic modalities. In response, the industry is actively exploring integrated continuous biomanufacturing, which combines a continuous upstream process with some or all downstream process operations (Arnold et al., 2019; Coffman et al., 2021; Coolbaugh et al., 2021; Natarajan et al., 2024; Warikoo et al., 2012). This shift from fed-batch to perfusion cell culture processes is essential for enabling continuous biomanufacturing, leading to stable operation with extended production times and increased productivity, while also improving product quality and reducing product residence times (Bielser et al., 2018; Chotteau, 2015). Recent efforts have focused on developing, optimizing, and improving perfusion processes for production cell lines (Bielser et al., 2019; Clincke et al., 2013a; Clincke et al., 2013b; Romann et al., 2023; Wolf et al., 2018; Wolf et al., 2019). Still, little attention has been given to the critical step of cell clone screening and selection of the lead clone. The aim of screening during cell line development is to narrow down thousands of individual cell line candidates (referred to as “cell clones”) to a single top clone that expresses the therapeutic protein with desired product quality attributes. The first step is typically carried out in microwell plates (MWP) at micro- and milli-litre scales to significantly reduce the number of clones before moving to screening in progressively larger small-scale bioreactors (12 mL – 3 L) and ultimately select the lead clone for GMP large-scale production. Perfusion mode is typically implemented at lab scale (>1 L). However, advancements in small-scale cell retention devices (i.e., hollow fibre membranes in tangential flow filtration (TFF) or alternating tangential flow filtration (ATF) mode) made it possible to implement perfusion processes at 250 mL scale. This reduces the required footprint and allows higher experimental throughput (Schwarz et al., 2020; Tregidgo et al., 2023a). In contrast, initial cell clone screening studies in MWPs typically use fed-batch operation as the standard process (Rouiller et al., 2016; Wang et al., 2018). In this mode, cells grow initially in basal medium, and additional bolus or continuous feeds are used to replenish nutrients using a highly concentrated feed medium to prolong growth and culture time (Xu et al., 2023). Perfusion mode is characterised by a continuous exchange of medium where the spent medium is continuously removed and replenished by fresh medium (Chen et al., 2018; Chotteau, 2015). This creates a more homogenous environment in contrast to fed-batch mode, where the product of interest and toxic by-products accumulate, resulting in a constantly changing environment (Chatterjee, 2012). Thus, the shift from high-nutrient feed steps to (semi-) continuous medium exchanges alters the physiological environment to which the cells are exposed, and could influence the overall cell performance during growth and production steps (Walther et al., 2019). This raises two pertinent questions: To what extent does the operation mode impact the cell clone ranking at early development stage? Does the use of fed-batch mode for the initial cell clone screening step lead to the exclusion of clones more suitable for perfusion process operation?

Some evidence from literature suggests that implementing the targeted operation earlier is beneficial for cell clone screening. Previous studies showed that an early stage fed-batch mode in well plates increases the predictability of performance at large scale (Wang et al., 2018), but can also impact the clone ranking (Markert et al., 2019). Markert et al. compared two cell clone screening procedures to identify the most suitable clone for fed-batch operation. The study showed that the automated process, which implemented the fed-batch operation earlier in the sequence, had an overall different clone ranking compared to the reference workflow. This automated workflow included clones that were considered “undesirable” from the reference workflow, and even identified a different top clone (Markert et al., 2019). So far only a limited number of studies have investigated cell clone screening in perfusion mode and the majority used spin tubes (ST) and ambr^®^15 bioreactor systems to conduct studies combining media and cell clone screening for process optimisation and scale up (Bielser et al., 2018; Gagliardi et al., 2019; Gomez et al., 2017). Bielser et al. investigated cell clone screening in deep-well plates (DWPs) and STs in semi-perfusion mode and found that both scale-down models were comparable regarding growth and productivity, and suitable for predicting appropriate process conditions in a perfusion bioreactor (Bielser et al., 2019). Despite the shown applicability of clone screening in semi-perfusion, and some studies investigating the transition of operation modes; a systematic comparison of cell clone screening in fed-batch and semi-perfusion mode has not yet been conducted.

Previous research extensively investigated a 24-well MWP in semi-perfusion mode where cell retention was achieved through centrifugation followed by medium exchange (Tregidgo et al., 2023b). These studies showed that high cell densities (<70 × 10^6^ cells mL^−1^) can be achieved and steady state can be successfully mimicked through cell bleeds, total or partial medium exchanges (Dorn et al., 2024a; Dorn et al., 2024b). Furthermore, the results showed comparability to a 5 L perfusion bioreactor (Tregidgo et al., 2023b).

In this work, we utilised the MWP platform to compare the impact of fed-batch and semi-perfusion mode on cell clone screening. For semi-perfusion mode, we investigated the effects of total and partial medium exchanges as well. Therefore, a panel of clonally-derived CHO cell lines producing a monoclonal antibody (mAb) were investigated and evaluated based on growth and metabolism. The clones were ranked using two strategies, the first considering a single parameter, the cell-specific productivity (q_p_), the second considered multiple parameters using the metric of Manufacturability Index (MI_CL_). Additionally, we evaluated a panel of clonally-derived CHO cell lines expressing a bispecific antibody (bspAb) to demonstrate the suitability of the methodology for different product cell lines.

## 2 Materials and methods

### 2.1 Cell culture, cell lines and media

Experiments were performed with two panels of proprietary clonally-derived Chinese hamster ovary (CHO) cell lines provided by AstraZeneca. The first CHO cell line panel expressed a proprietary monoclonal antibody (mAb1) and comprised eight clones (mAb1_C1 – C8), whilst the second CHO cell line panel expressed a proprietary bispecific antibody (bspAb1) and comprised six clones (bspAb1_C1 – C6). Cells were cultivated in two commercially available media: (1) fed-batch specific CD CHO medium (Gibco^®^, Thermo Fisher Scientific, Massachusetts, United States) and (2) perfusion-specific High Intensity Perfusion (HIP) medium (Gibco^®^, Thermo Fisher Scientific, Massachusetts, United States), both supplemented with 4% 50X HT supplement (Gibco^®^, Thermo Fisher Scientific, Massachusetts, United States). Cell banks were established for each cell clone and medium prior to the clone screening experiments. Cell suspensions were cultivated in 125 mL non-baffled shake flasks (Corning^®^, United States) placed in an incubator (MCO-19AIC, Sanyo, JP) at 37°C, 5% CO_2_, and were agitated at a shaking speed of 180 rpm, using an orbital shaker with an orbital diameter (OD) of 25 mm (CO_2_ resistant shaker, Thermo Fisher Scientific). Cells were passaged every 3–4 days and expanded into 500 mL non-baffled shake flasks (Corning^®^, United States) for use for inoculation.

### 2.2 Process operations–microwell plate cultures

MWP cultures were performed using standard round well ultra-low attachment 24-well MWPs (CLS3473, Corning^®^). The MWPs were sealed with a Duetz sandwich lid (CR1524, EnzyScreen, Heemstede, NL) to reduce evaporation while maintaining headspace gas exchange. All cultures were cultivated in an incubator at 37°C, 5% CO_2_, an agitation speed of 250 rpm, and an OD of 19 mm, unless otherwise indicated. The plates were held in place by a Duetz MWP clamp system (CR1801h, EnzyScreen, Heemstede, NL).

For fed-batch (FB) cultures, MWPs were inoculated at 1 × 10^6^ cells mL^−1^ at a working volume of 1.2 mL in CD CHO medium (day 0). Following a 3-day batch phase, feeding commenced on day 3 for a period of 5 consecutive days. Feeding was conducted as previously described by Silk et al., 2010 and involved a 6% v/v bolus addition of nutrient supplement (EfficientFeed™ B), followed with a 2.5% v/v bolus of 10x diluted bicarbonate solution (0.75 M Na_2_CO_3_, 0.5 M NaHCO_3_) to control pH. For semi-perfusion (SP) cultures, MWPs were inoculated at 1 × 10^6^ cells mL^−1^ at a working volume of 1.2 mL (day 0) in CD CHO or HIP medium. Following a batch phase, semi-perfusion was started on day 3, where total or partial medium exchanges equal to 1 RV d^−1^ and 0.75 RV d^−1^, respectively, were performed. A sacrificial well methodology was used for both process operations, where triplicate samples were taken every 24 h from day 3 to day 10.

### 2.3 Process analytics

Viable cell concentrations (VCCs) and percentage of viability were determined using a ViCell™ XR cell viability analyser (Beckman Coulter, United States). Due to the small sample size for MWP cultures, the number of extracellular metabolites was limited to three. Glucose, lactate, and ammonium were measured using an Optocell CuBiAn VC biochemistry analyser (4BioCell, Bielefeld, Germany) as described in Dorn et al. (2024a) and Dorn et al. (2024b). Osmolality was determined using a freeze point osmometer (Gonotec^®^ Osmomat 3000).

For titre quantification, an HPLC (HPLC Agilent 1100 series; Agilent, United States) with a 1 mL Protein G column (HiTrap™ Protein G HP, Cytiva) was used with loading buffer A (20 mM phosphate, pH 7.0) and elution buffer B (20 mM glycine, pH 2.8) to evaluate the protein concentration. An IgG standard with a concentration of 1.9 g L^−1^ was used for both cell lines (determined by Nanodrop 1000, Labtech). The IgG standard was diluted in PBS and measured in triplicate to obtain a standard curve in range of 0–1.9 g L^−1^.

For comparative analysis between different operation modes and perfusion rate strategies, the following Equations 1–4 were used to determine cell specific rates, and yields.

$$q=\left(\frac{∆c}{∆t\cdot \bar{X}}\right)+\frac{\left(H+B\right)\cdot c_{i}}{\bar{X}}$$ (1)

$$STY=\frac{Y_{i}}{V_{W}\cdot \left(t_{i}-t_{0}\right)}$$ (2)

$$Y_{i}=\int_{0}^{i}c_{P,i}\cdot H_{i}\cdot V_{W}\;dt$$ (3)

$$Y_{P/Gluc}=\left(\frac{{STY}_{i}\cdot ∆t}{c_{Gluc,0}-c_{Gluc,i}}\right)\frac{1}{V_{w}}$$ (4)

where *q* is the cell specific consumption/production rate, *H* is the daily harvest rate, *B* is the daily bleed rate, Δt is the time interval between two sampling time points, $\bar{X}$ is the daily average of the VCC, and *c* is the metabolite or product concentration, *STY* is the space-time-yield, *Y*_*i*_ the yield equal to the accumulated mass produced since start of the cultivation, *Y*_*P/Gluc*_ the product-over-glucose yield, *c*_*P*_ the antibody concentration at time *i*, *c*_*Gluc*_ the glucose concentration at time *i*, *V*_*W*_ the working volume, and *t* the cultivation time.

### 2.4 Manufacturability index (MI_CL_)

The cell line manufacturability index (MI_CL_) leverages available data on growth characteristics, metabolites, and productivities by combining these into a single metric for the evaluation of *m* cell lines according to *n* criteria (Goldrick et al., 2023). The selection of the lead clone is formulated as a multi-criteria decision-making problem to allow assessing the performance of each individual clone (Goldrick et al., 2023). In this work, the MI_CL_ was adapted to MWP operation and for the comparison of fed-batch and semi-perfusion operations. To achieve a fair comparison with an equal number of parameters and to account for the differences in feeding and medium exchange protocols, the following parameters and criteria were selected: maximum values of viable cell concentration (VCC), growth rate, space-time-yield (STY) and product-over-glucose yield, minimum values of viability, and lactate concentration, as well as average values of cell specific production and consumption rates of the product and metabolites (q_P_, q_Gluc_, q_Lac_, q_Amm_). The parameters and criteria were fixed in this work, but the selection could be adjusted according to individual user requirements.

The calculation and data visualisation were performed in MATLAB 2021b based on the following equations previously described by Goldrick et al., 2023, where the MI_CL_ was calculated using Equation 5.

$${MI}_{CL,i}=\sum_{j=1}^{j=n}w_{j}\times r_{ij}\;for\;i=1,2,3,\ldots m$$ (5)

Where *w*_*i*_ is the normalised weight of each criteria *j*, *r*_*ij*_ is a dimensionless rating per clone *i* and selection criteria *j*:

$$r_{ij}=\left|\frac{x_{ij}-x_{j,worst}}{x_{j,best}-x_{j,worst}}\right|$$ (6)

In Equation 6 *x*_*ij*_ is the individual ranking of cell clone *i* for criteria *j*, *x*_*j,best*_ is the best overall ranking, *x*_*j, worst*_ is the worst overall ranking for criteria *j*. *x*_*j,best*_ and *x*_*j, worst*_ were defined as maximum or minimum values based on expertise from industry, where some parameters are considered “best” for the maximum value and “worst” for the minimum value of the parameter *j* (e.g., VCC or q_P_) while others have a reverse rating where the minimum value is considered “best” and the highest value considered “worst” (e.g., lactate concentrations). The normalised weights were set to 1 for this study but could be adjusted in the future or depending on individual process requirements.

## 3 Results

The cell clone screening experiments involved two antibody producing CHO cell lines (mAb1, bspAb1) and a total of 14 clones. The clones for mAb1 were used as a base case study and additional experiments were conducted using clones for bspAb1 to demonstrate applicability across different cell lines and protein products. Experiments are referred to as FB for fed-batch, SP-CD CHO and SP-HIP for semi-perfusion in CD CHO and HIP at 1 RV d^−1^, respectively.

### 3.1 Growth performance and metabolism

#### 3.1.1 Fed-batch vs. semi-perfusion comparison

**FIGURE 1:**
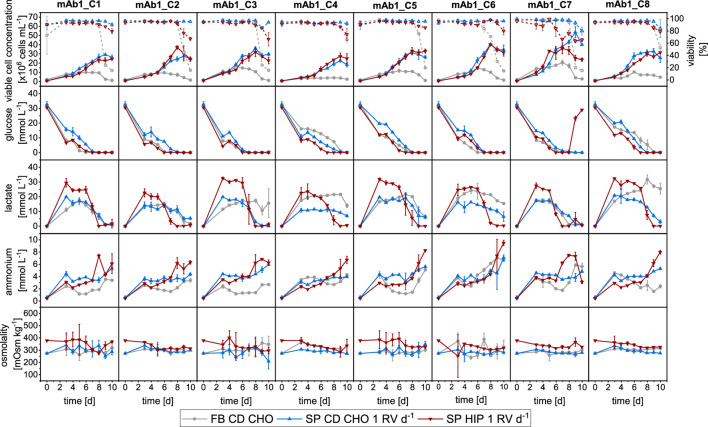
Overview of growth and metabolites for mAb1 CHO cell clones in MWPs using fed-batch and semi-perfusion methodologies. Cells were inoculated at 1 × 10^6^ cells mL^−1^ for all methodologies. For fed-batch cultures, cells were cultivated in CD CHO with a feed step from day 3 to day 7 (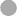). For semi-perfusion cultures, a perfusion rate of 1 RV d^1^ was used from day 3 to day 10 and cells were cultivated in CD CHO (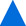) and HIP media (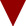). Row 1: viable cell concentration (closed, straight) and viability (open, dashed), Row 2: glucose concentration; Row 3: lactate concentration; Row 4: ammonium concentration; Row 5: osmolality. Columns display the eight individual clones. Mean of N = 3 wells. Error bars indicate standard deviation.

First, the growth performance and metabolism of the mAb1 cell clones were investigated using MWP methodologies in FB and SP with a perfusion rate equal to 1 RV d^−1^, typically used for manual SP operation, and two media. Figure 1 shows the growth performance, as well as measurements of external metabolites and osmolality for all eight clones producing mAb1.

All eight clones grew as expected using both methodologies with SP cultures achieving higher VCCs than FB cultures (Figure 1, Row 1). While FB cultures reached maximum VCCs in range of 4–21 × 10^6^ cells mL^−1^ on days 5–7, SP culture in both CD CHO and HIP medium reached maximum values of 23–54 × 10^6^ cells mL^−1^ and 25–41 × 10^6^ cells mL^−1^, respectively. The highest maximum VCC was obtained with mAb1_C7 in all three experiments. Viabilities were above 95% for all clones during growth phase but started to decrease from day 6 for FB cultures dropping below 50% on day 10, except for mAb1_C4 which had a viability around 70% on day 10. SP-CD CHO cultures remained above 90% throughout the cultivation duration, while for HIP-adapted cultures the viability started to decrease on day 8 and dropped to values of 60%–80% on day 10 (Figure 1, Row 1).

Metabolite concentrations of glucose, lactate, and ammonium are shown in Figure 1 in Row 2, 3 and 4, respectively. Both media (CD CHO and HIP) had similar concentrations of glucose at the start of cultivation around 30 mmol L^−1^. All cultures showed a decrease of glucose concentrations towards depletion between days 6 and 10 (Figure 1: Row 2), whereby SP cultures depleted glucose slightly earlier than FB cultures. For all clones, methods, and media combinations, the glucose concentration was 0 mmol L^−1^ on day 10. An exception was mAb1_C7 in SP-HIP, where the glucose concentrations increased on days 9 and 10, which coincided with a decrease of VCC and viability.

Lactate concentrations showed greater differences between operation modes and media than glucose (Figure 1: Row 3). Clones cultivated in CD CHO medium for both FB and SP modes showed similar dynamics, where nearly identical lactate concentration profiles were obtained. The profile showed an initial increase to concentrations in range of 15–20 mmol L^−1^, which remained stable for 3–4 days followed by a slow decrease towards the end of the culture. Some clones (e.g., mAb1_C4) obtained slightly higher lactate concentrations of around 25 mmol L^−1^ for FB but showed otherwise similar dynamics. For mAb1_C3 and mAb1_C8 greater differences were observed between operation modes. After an initial increase, the lactate concentrations decreased for SP-CD CHO cultures whilst remaining stable for FB cultures. In contrast to FB and SP-CD CHO cultures, all clones in SP-HIP showed an initial increase to lactate concentrations in range 15–25 mmol L^−1^ which remained stable for 3–4 days followed by a decrease towards the end of culture with depletion on the last 2 days. The decrease of lactate for all cultures coincided with a depletion of glucose of the respective cultures thus indicating a shift from lactate production to consumption. Ammonium concentrations increased over time for all operation modes and cultures (Figure 1: Row 4). However, ammonium concentrations were generally lower for cultures in CD CHO medium than for cultures in HIP medium and remained stable for several days. SP-HIP cultures obtained higher concentrations with a pronounced increase towards the end of culture, which coincided with a reduction in lactate. Overall, endpoint ammonium concentrations were in range of 3–8 mmol L^−1^ for cultures in CD CHO and 6–10 mmol L^−1^ for SP-HIP cultures.

The osmolality was stable throughout the culture duration for all experiments, however, was slightly higher for SP-HIP cultures (330–370 mOsm kg^−1^, Figure 1: Row 5). Cultures in CD CHO had an osmolality in range of 270–320 mOsm kg^−1^ (Figure 1: Row 5).

To summarise, a comparative analysis of fed-batch and semi-perfusion cultivation showed distinct cell growth patterns, with clones cultured under semi-perfusion conditions consistently achieving higher VCCs than those in fed-batch mode. Although glucose, ammonium, and osmolality levels exhibited comparable trends across both methods and media, lactate concentration dynamics demonstrated pronounced differences between the two cultivation strategies and media types.

#### 3.1.2 Perfusion rate comparison

After understanding the impact of operation mode, the clones were investigated in SP with different perfusion rates. This involved comparing a total medium exchange (1 RV d^−1^) to a partial medium exchange (0.75 RV d^−1^). The goal was to investigate potential differences in clone performance between a typical manual SP operation with 1 RV d^−1^ and prospective automated workflow that would use a partial medium exchange due to the operation with a liquid handling arm and the use of sedimentation as the cell retention method. It should be noted that the same SP-HIP dataset as in Section 3.1 was used for comparison with the partial medium exchange, which will be referred to as SP-HIP-75%.

**FIGURE 2:**
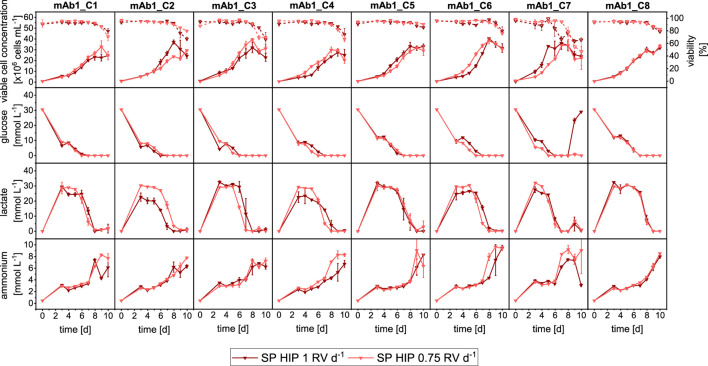
Overview of growth and metabolites for mAb1 CHO cell clone screening in MWPs using semi-perfusion methodologies with different fixed perfusion rates. Cells were inoculated at 1 × 10^6^ cells mL^−1^ and cultivated in HIP medium with a perfusion rate of 1 RV d^−1^ (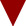) and 0.75 RV d^−1^ (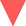). Row 1: viable cell concentration (closed, straight) and viability (open, dashed), Row 2: glucose concentration; Row 3: lactate concentration; Row 4: ammonium concentration. Columns display the eight individual clones. Mean of N = 3 wells. Error bars indicate standard deviation.

The comparison of SP-HIP cultures for all clones with the two different perfusion rates is shown in Figure 2. Overall, all clones grew similarly at the two perfusion rates. Some differences of note were observed for clones mAb1_C1 and mAb1_C3, which achieved slightly higher maximum VCCs with the partial medium exchange (mAb1_C1: 31 × 10^6^ cells mL^−1^ and mAb1_C3: 40 × 10^6^ cells mL^−1^) compared to the total medium exchange (mAb1_C1: 25 × 10^6^ cells mL^−1^ and mAb1_C3: 33 × 10^6^ cells mL^−1^). In contrast, mAb1_C2 and mAb1_C5 achieved marginally higher maximum VCCs with the total medium exchange. The differences observed were not significant and maximum VCCs were in range of 28–38 × 10^6^ cells mL^−1^ for SP-HIP-75% cultures and 27–40 × 10^6^ cells mL^−1^ for SP-HIP cultures. Similar observations can be made for all cell viabilities, where for all clones the viabilities remained above 95% until day 8 and decreased to values of 60%–80% on day 10, with marginal to no differences between the results obtained at the two perfusion rates. An exception is mAb1_C7 where for SP-HIP the viability started to decrease already from day 6, while for the SP-HIP-75% the viability decreased from day 8. However, on day 10, for both experiments, viabilities were within the range of error and around 60%.

In agreement with the growth dynamics, the metabolite profiles also showed similarities (Figure 2: Row 2–4) between both medium exchanges. It is interesting to note that for the majority of clones the glucose and lactate concentrations show very similar concentration levels and dynamics. It was expected to observe a faster reduction and depletion of glucose concentrations for SP-HIP-75% cultures than for SP-HIP cultures. However, only clones mAb1_C4 and mAb1_7 of SP-HIP-75% cultures showed an earlier onset of glucose consumption with depletion on day 6 and 5 respectively, while for the total medium exchange glucose was depleted 1 day later. For lactate concentration, it was expected to obtain generally higher concentration for SP-HIP-75% cultures due to accumulation, which was only the case for mAb1_C2 and mAb1_C4 with notable differences of lactate concentrations up to 30 mmol L^−1^ for SP-HIP-75% and 20–25 mmol L^−1^ for SP-HIP for a duration of 3–4 days. The clones mAb1_C6 and mAb1_C7 also obtained higher lactate concentration, however, only for 2–3 days with concentrations in range of 20–25 mmol L^−1^ for both medium exchange regimes. Overall, the concentration dynamic was identical for all clones between the different perfusion rates, where concentrations initially increased and plateaued at stable values before starting to decrease around days 6–7, with depletion towards the end of culture on day 10.

Ammonium concentrations increased throughout the cultivation duration for all clones, with a sudden increase around day 7, which coincided with the depletion of glucose and a drastic reduction of lactate. The concentrations reached on day 10 were in range of 6–10 mmol L^−1^ for both perfusion rates.

In summary, a comparison of growth and metabolic performance between clones cultured under semi-perfusion conditions with total and partial medium exchange showed substantial similarities. While some clones demonstrated marginally higher VCCs under partial medium exchange, these differences were not statistically significant.

### 3.2 Clone ranking

#### 3.2.1 Single-parameter clone ranking–cell specific productivity

A cell clone ranking analysis was initially performed based on a single parameter–the cell-specific productivity (q_P_) – for all FB and SP cultures previously described in Sections 3.1.1 and 3.1.2. In Table 1, the q_P_ based clone rankings are presented, where a colour code was employed, assigning a colour to each clone ranging from violet (mAb1_C1) to dark red (mAb1_C8). This aims to simplify the ranking visually. In addition, the raw q_P_ values are reported in Supplementary Table S1 in the Supplementary Material, and are in range of 1.64–25.47, 17.31–41.95, 16.46–39.14 and 16.46–46.57 pg cell^−1^ d^−1^ for FB, SP-CD CHO, SP-HIP and SP-HIP-75%, respectively. All experiments in SP mode obtained a similar range for q_P_ values, while q_P_ values were 2 – 8-fold lower for FB. It should be noted that the ranking was performed for each experiment individually, and q_P_ values are evaluated within reference of the individual range of the experiment.

**TABLE 1:** Ranking of eight mAb1 CHO cell clones based on average cell specific productivity values for fed-batch and semi-perfusion operation with total and partial medium exchanges in CD CHO and HIP medium.

Ranking position	FB		SP-CD CHO		SP-HIP		SP-HIP-75%	
	—		1 RV d^−1^		1 RV d^−1^		0.75 RV d^−1^	
#1	mAb1_C4		mAb1_C4		mAb1_C4		mAb1_C5	
#2	mAb1_C5		mAb1_C6		mAb1_C6		mAb1_C4	
#3	mAb1_C8		mAb1_C1		mAb1_C1		mAb1_C2	
#4	mAb1_C2		mAb1_C5		mAb1_C5		mAb1_C1	
#5	mAb1_C1		mAb1_C2		mAb1_C2		mAb1_C8	
#6	mAb1_C6		mAb1_C7		mAb1_C3		mAb1_C7	
#7	mAb1_C3		mAb1_C3		mAb1_C8		mAb1_C6	
#8	mAb1_C7		mAb1_C8		mAb1_C7		mAb1_C3	

Colour code to simplify the ranking visually. For SP cultures the average was calculated from day 3 to day 10.

The clone ranking between operation modes (FB vs. SP) showed large differences, while for SP-CD CHO and SP-HIP only minor differences were obtained at low-ranking positions (#6 – #8; Table 1). The same clone mAb1_C4, was identified as top performer for both operation modes, however, there were significant differences in other ranking positions. For example, mAb1_C6 and mAb1_C1 rank at position #2 and #3 in SP respectively (in SP q_P_ was in range of 16.46–41.95 pg cell^−1^ d^−1^ for both media with mAb1_C6: 30.1–33.1 pg cell^-1^ d^-1^ and mAb1_C1: 28.9–29.7 pg cell^−1^ d^−1^), while in FB both clones rank much lower, at positions #6 and #5 (in FB q_P_ ranged was in range of 1.64–25.47 pg cell^−1^ d^−1^ with mAb1_C6: 5.40 pg cell^−1^ d^−1^ and mAb1_C1: 6.08 pg cell^−1^ d^−1^). Clone mAb1_C8 ranked third in FB (in FB q_P_ ranged was in range of 9.55 pg cell^−1^ d^−1^) but shared the last two positions in SP (#7 and #8; q_P_: 16.58 and 17.31 pg cell^−1^ d^−1^ for SP-HIP and SP-CD CHO, respectively). However, it must be noted that some clones shared similar trends across methods such as mAb1_C2, which was ranked at middle positions #4 and #5, or mAb1_C3 which was among the last 3 ranking positions in all cases (Table 1).

Comparison of the clone ranking in SP performed at different perfusion rates (1 RV d^−1^ vs. 0.75 RV d^−1^), showed differences even though the obtained q_P_ values ranged similarly with values of 16.46–39.14 and 16.46–46.57 pg cell^−1^ d^−1^ for SP-HIP and SP-HIP-75%, respectively. Although, mAb1_C4 shared the top 2 positions in both SP-HIP and SP-HIP-75% (39.14 and 32.14 pg cell^−1^ d^−1^, respectively), the top performer for SP-HIP-75% was only at #4 for SP-HIP (mAb1_C5; with a q_P_ of 29.45 pg cell^−1^ d^−1^ for SP-HIP and 46.57 pg cell^−1^ d^−1^ for SP-HIP-75%). The second best clone of SP-HIP (mAb1_C6; 33.06 pg cell^−1^ d^−1^) ranked at #7 in SP-HIP-75% (22.13 pg cell^−1^ d^−1^). Nonetheless, a trend of clones ranking in similar low (e.g., mAb1_C3), middle (e.g., mAb1_C1) or high (e.g., mAb1_C4) positions was observed.

In summary, the ranking of clones based solely on the q_P_ parameter revealed a shared top clone between FB and SP with total medium exchange (1 RV d^−1^). However, disparities emerged in the subsequent clone rankings when comparing FB and SP with total medium exchange, and similarly for the comparison of clone rankings for SP with total and partial medium exchanges. These findings suggest that the methodology employed and the perfusion rate utilized in SP can substantially influence the clone ranking outcome.

#### 3.2.2 Multi-parameter clone ranking–manufacturability index

The previous analysis using a single parameter offered valuable insights but was limited by neglecting other crucial parameters such as growth and metabolites. To address this limitation, the clone selection is formulated as a multi-criteria decision-making problem based on a strategy previously introduced by Goldrick et al. (2023). This approach was adapted for the MWP scale and tailored for comparing different operational modes, incorporating a broader range of parameters, including growth, metabolism and other productivity metrics alongside q_P_ (see Section 2.4). These parameters were integrated to create a single metric known as the Cell line Manufacturability Index (MI_CL_) to assess the clone ranking. The raw MI_CL_ data is reported in Supplementary Table S1 in the Supplementary Material, with values in range of 0.14–1.00, 0.62–1.00, 0.38–1.00 and 0.43–1.00 for FB, SP-CD CHO, SP-HIP and SP-HIP-75%, respectively. It should be noted that the maximum possible value of the MI_CL_ is 1 due to a normalisation during the calculation (see Section 2.4). Thus, the MI_CL_ are evaluated within reference of the individual range of the experiment. The range of MI_CL_ for FB were larger than for SP, where SP-HIP and SP-HIP-75% had a similar range and SP-CD CHO had the narrowest range of MI_CL_ values.

**FIGURE 3:**
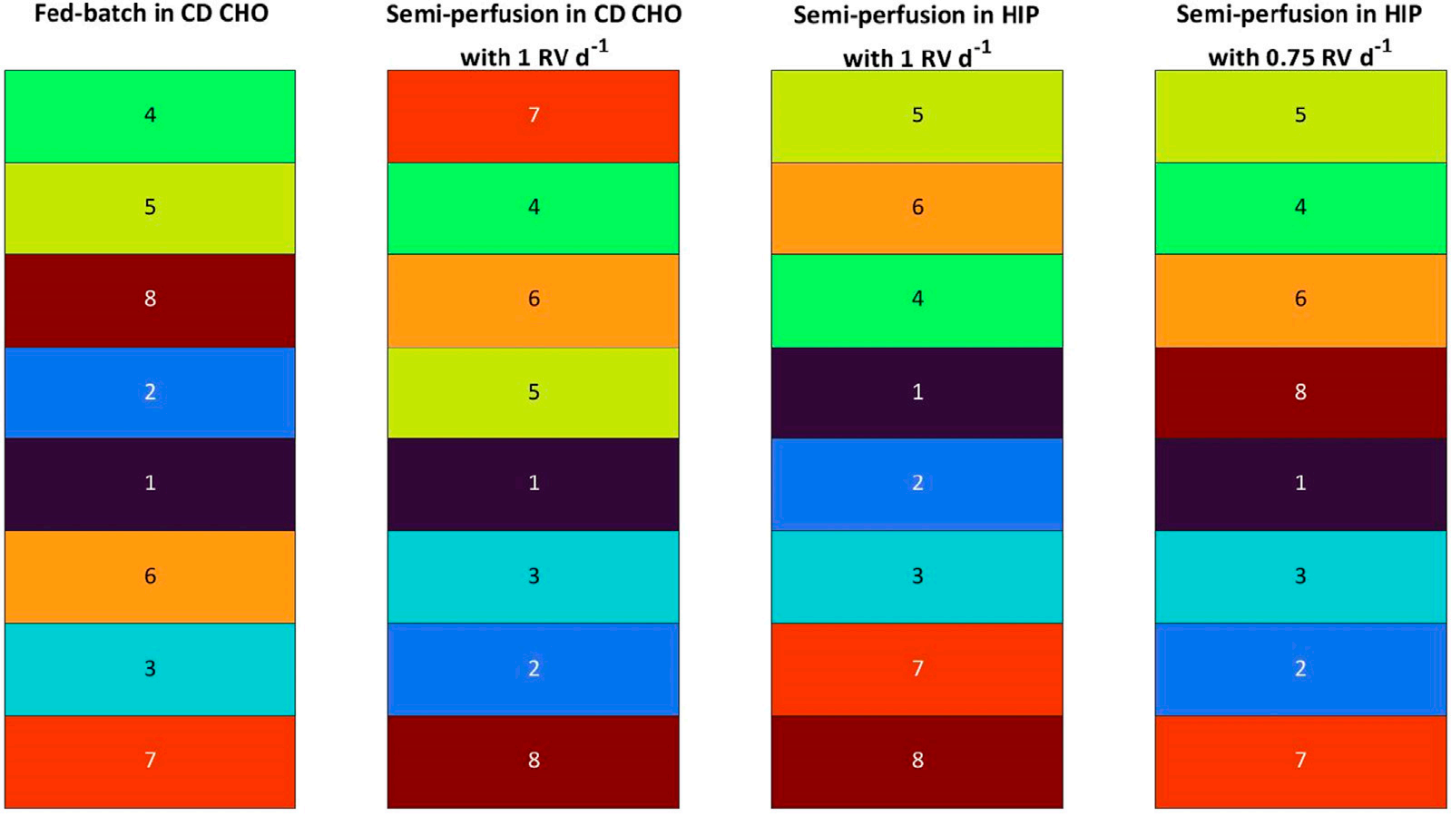
Ranking of eight mAb1 CHO cell clones based on Manufacturability index for fed-batch and semi-perfusion operation with total and partial medium exchanges in CD CHO and HIP medium.

In Figure 3, the MI_CL_ based ranking is presented, where the same colour code as for the q_P_ strategy was employed assigning a colour to each clone ranging from violet (mAb1_C1) to dark red (mAb1_C8). Comparing the clone ranking of FB and SP with 1 RV d^-1^ in both media, significant differences are observed between both operation modes and media. While some minor ranking differences between SP-CD CHO and SP-HIP were observed, the same clones grouped on top, middle or worse positions for SP in both media. As an example, mAb1_C8 ranked at position #8 for both media (MI_CL_: 0.61 for SP-CD CHO and 0.38 for SP-HIP, with the MI_CL_ in range of 0.61–1.00 and 0.38–1.00, respectively) or clones mAb1_C4 and mAb1_C6 shared ranks #2 and #3. The exception was mAb1_C7 which ranked significantly better at #1 for SP-CD CHO (MI_CL_; 1.00 (0.61–1.00)) compared to #7 for SP-HIP (MI_CL_: 0.42 (0.38–1.00)). Comparing FB and SP larger differences were observed. Although the top clone for FB (mAb1_C4) is among the top three positions for SP, mAb1_C8, which ranked #3 for FB (MI_CL_: 0.54 (0.14–1.00)) ranked last for SP (MI_CL_: 0.61 for SP- CD CHO (0.61–1.00) and 0.38 for SP-HIP (0.38–1.00)). Further mAb1_C2 ranked slightly higher for FB than for SP, while mAb1_C6 ranked generally lower in FB (#6) than in SP (#3, #2).

In contrast, the ranking of SP-HIP and SP-HIP-75% showed fewer differences, where for both conditions the top clone was identical (mAb1_C5). The positions #2 and #3 were shared by the same clones mAb1_C4 (MI_CL_: 0.89 and 0.78 for SP-HIP-75% and SP-HIP, respectively) and mAb1_C6 (MI_CL_: 0.75 and 0.80 for SP-HIP-75% and SP-HIP, respectively), while mAb1_C7 was among the last two positions. The exception was clone mAb1_C8 with ranked last for SP-HIP (MI_CL_: 0.38 (0.38–1.00)) but fourth for SP-HIP-75% (MI_CL_: 0.69 (0.43–1.00)).

Interestingly, mAb1_C1 and mAb1_C3 ranked nearly the same on middle and low positions regardless the operation mode, medium or perfusion rate used.

Comparing across the ranking strategies by q_P_ (Table 1) and MI_CL_ (Figure 3), it can be observed that the clone ranking for FB was identical between the two strategies, whereas for SP much greater differences were present. SP-CD CHO showed the greatest differences with the most prominent position changes over multiple ranks, where mAb1_C7 was ranked at #1 for the MI_CL_ strategy (MI_CL_: 1.00 (0.61–1.00)) instead of #6 for the q_P_ strategy (q_P_: 19.64 pg cell^−1^ d^−1^ (17.31–41.95 pg cell^−1^ d^−1^)) while mAb1_C2 ranked at #5 and #7 for q_P_ and MI_CL_, respectively (q_P_: 24.03 pg cell^−1^ d^−1^ (17.31–41.95 pg cell^−1^ d^−1^); MI_CL_: 0.69 (0.61–1.00)). In contrast, SP-HIP showed only minor differences where clones changed by only one or two positions, besides mAb1_C5 which ranked at #4 for the q_P_ strategy (q_P_: 29.45 pg cell^−1^ d^−1^ (16.46–39.14 pg cell^−1^ d^−1^)) and #1 for MI_CL_. (MI_CL_: 1.00 (0.38–1.00)). Lastly, for SP-HIP-75% the same top two clones (mAb1_C5 and mAb1_C4) were obtained for both q_P_ and MI_CL_ ranking strategies, while mAb1_C2 and mAb1_C6 swapped ranking positions from better to worse for q_P_ and MI_CL_, respectively.

To contextualise, the MI_CL_ ranking, incorporating multiple parameters, corroborated the hypotheses derived from the q_P_-based ranking. In contrast to FB, SP systems with varying media and perfusion rates exhibited substantial differences in clone rankings. Nevertheless, a discernible trend emerged across all SP rankings, indicating a tendency for certain clones to consistently occupy high or low positions. Furthermore, a comparative analysis of q_P_ and MI_CL_ ranking strategies revealed identical rankings for FB but divergent results for SP.

### 3.3 Reproducibility

While the previous investigations showed the impact of the operation mode on growth, metabolism and clone ranking, a crucial step is to ensure the reproducibility of these results. Therefore, an investigation was conducted to assess the reproducibility of growth performance and ranking results. A second run of SP-HIP-75% was performed, with cells thawed from a second vial for each clone and compared to the SP-HIP-75% described in Section 3.1.2, now referred to as Run 1.

**FIGURE 4:**
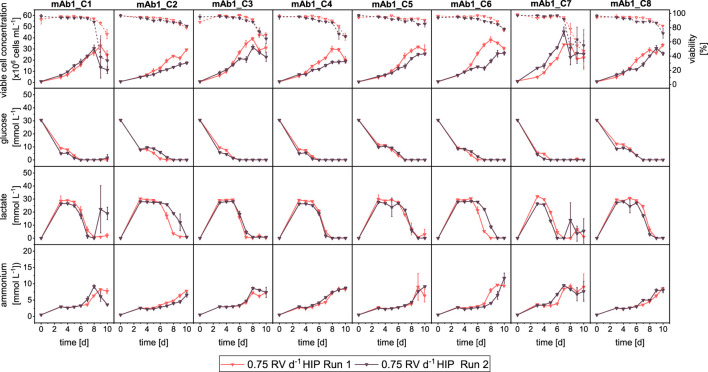
Overview of growth and metabolites for mAb1 CHO cell clone screening in MWPs using semi-perfusion methodologies. Cells were inoculated at 1 × 10^6^ cells mL^−1^ and cultivated in HIP medium with a perfusion rate of 0.75 RV d^−1^ Run 1 (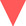) and 0.75 RV d^−1^ Run 2 (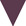). Row 1: viable cell concentration (closed, straight) and viability (open, dashed), Row 2: glucose concentration; Row 3: lactate concentration; Row 4: ammonium concentration. Columns display the eight individual clones. Mean of N = 3 wells. Error bars indicate standard deviation.

The growth performance and external metabolites of both runs are shown in Figure 4 and in general showed similar dynamics. In particular, metabolite concentrations were nearly identical between both runs (Figure 4: Row 2–4), where only for two clones (mAb1_C2 and mAb1_C6) some differences of lactate concentrations were observed, with a delayed onset of the reduction of lactate concentration. However for the growth performance, larger differences between Run 1 and Run 2 were observed (Figure 4: Row 1). The maximum VCCs of Run 2 were lower than those of Run 1. For example, mAb1_C2 reached 29.44 × 10^6^ cells mL^−1^ and 17.51 × 10^6^ cells mL^−1^ in Run 1 and 2, respectively. An exception was mAb1_C7, which reached around 45 × 10^6^ cells mL^−1^ in Run 2 in contrast to around 35 × 10^6^ cells mL^−1^ in Run 1.

The clone ranking was evaluated for a single parameter strategy as well as with the MI_CL_ strategy. The raw values of q_P_ ranging from 16.46–46.57 pg cell^−1^ d^−1^ for Run 1 and from 12.21–40.98 pg cell^−1^ d^−1^ for Run 2 and MI_CL_ ranging from 0.43–1.00 for Run 1 and 0.43–1.00 for Run 2 are reported in Supplementary Table S1. The ranges of q_P_ and MI_CL_ for both runs were similar.

**FIGURE 5:**
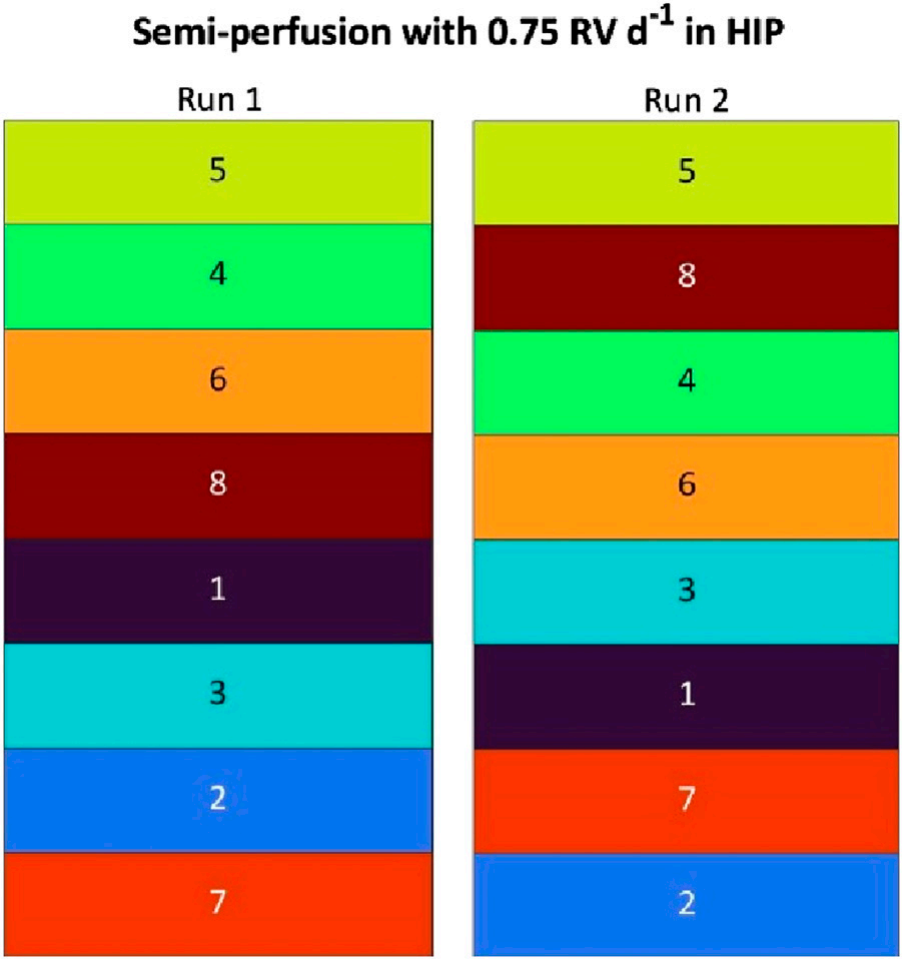
Ranking of eight mAb1 CHO cell clones based on Manufacturability index for semi-perfusion operation with partial medium exchanges in HIP medium.

The clone ranking based on the q_P_ is shown in Table 2. Both runs shared the same two top clones (#1: mAb1_C5 with q_P_: 46.57 and 40.98 pg cell^−1^ d^−1^ for Run 1 and 2 respectively; #2: mAb1_C4 with q_P_: 32.14 and 35.13 pg cell^−1^ d^−1^ for Run 1 and 2, respectively) as well as the same lowest-ranking position (#8, mAb1_C3), while the middle ranking position showed some variation with several clones changing by multiple positions. For example, mAb1_C6 ranked at positions #7 (q_P_: 22.13 pg cell^−1^ d^−1^) and #4 (28.77 pg cell^−1^ d^−1^) for Run 1 and Run 2, respectively, or mAb1_C2 ranked at #3 in Run 1 (q_P_: 27.58 pg cell^−1^ d^−1^) and #5 in Run 2 (q_P_:23.66 pg cell^−1^ d^−1^). However, when taking multiple parameters into account (Figure 5; based on MI_CL_), the ranking of Run 2 showed that ranking positions differed only by one position relative to Run 1. In both runs the same clone mAb1_C5 was identified as top clone (#1). Further, the positions #7 and #8 (mAb1_C2 (MI_CL_: 0.63 and 0.43) and mAb1_C7 (MI_CL_: 0.43 and 0.54)) as well as #5 and #6 (mAb1_C1 (MI_CL_: 0.69 and 0.57) and mAb1_C3 (MI_CL_: 0.68 and 0.65)) were shared by the same clones. Only for mAb1_C8, a greater change by 2 positions was observed, where for Run 1 it ranked at position #4 (MI_CL_: 0.69 (0.43–1.00)) and for Run 2 it ranked at #2 (MI_CL_: 0.86 (0.43–1.00)).

**TABLE 2:** Ranking of eight mAb1 CHO cell clones based on average cell specific productivity values for semi-perfusion operation with partial medium exchanges in HIP medium.

Ranking position	SP-HIP-75%		SP-HIP-75%	
	Run 1		Run 2	
#1	mAb1_C5		mAb1_C5	
#2	mAb1_C4		mAb1_C4	
#3	mAb1_C2		mAb1_C8	
#4	mAb1_C1		mAb1_C6	
#5	mAb1_C8		mAb1_C2	
#6	mAb1_C7		mAb1_C7	
#7	mAb1_C6		mAb1_C1	
#8	mAb1_C3		mAb1_C3	

Colour code to simplify the ranking visually. For SP, cultures the average was calculated from day 3 to day 10.

Moreover, comparing across ranking methods from Table 2 (based on q_P_) and Figure 5 (based on MI_CL_), it can be seen that for both methods the same clone mAb1_C5 ranked at #1 (q_P_: 46.57 and 40.98 pg cell^−1^ d^−1^) and mAb1_C4 was among the top 3 candidates (q_P_: 32.14 and 35.13 pg cell^−1^ d^−1^; MI_CL_: 0.89 and 0.83). However, some clones ranked considerably worse when using the MI_CL_ than when using the q_P_ strategy. For example, mAb1_C2 ranked at #7 and #8 for the MI_CL_ strategy but at middle positions (#3, #5) for q_P_. Similarly for mAb1_C3, with positions #5 and #6 for MI_CL_ (0.68 and 0.65 (0.43–1.00 and 0.44–1.00)), but at position #8 for the q_P_ based ranking strategy (q_P_: 20.18 and 18.81 pg cell^−1^ d^−1^ (16.46–46.57 and 12.21–40.98 pg cell^−1^ d^−1^)), while for mAb1_C6 the reverse was observed, ranking better using the MI_CL_ at #3 and #4 (0.75 and 0.746 (0.43–1.00 and 0.43–1.00)) than the q_P_ based strategy at #7 and #4 (q_P_: 22.13 and 28.77 pg cell^−1^ d^−1^ (16.46–46.57 and 12.21–40.98 pg cell^−1^ d^−1^)).

To evaluate reproducibility, two independent SP-HIP-75% experiments were conducted, demonstrating comparable growth and metabolite dynamics. The q_P_-based ranking yielded identical top two clones but exhibited some variability in lower-ranked positions. In contrast, the MI_CL_-based ranking identified the same top clone with minimal positional shifts in subsequent rankings, resulting in greater overall consistency across the two runs.

### 3.4 bspAb1 cell clones

**FIGURE 6:**
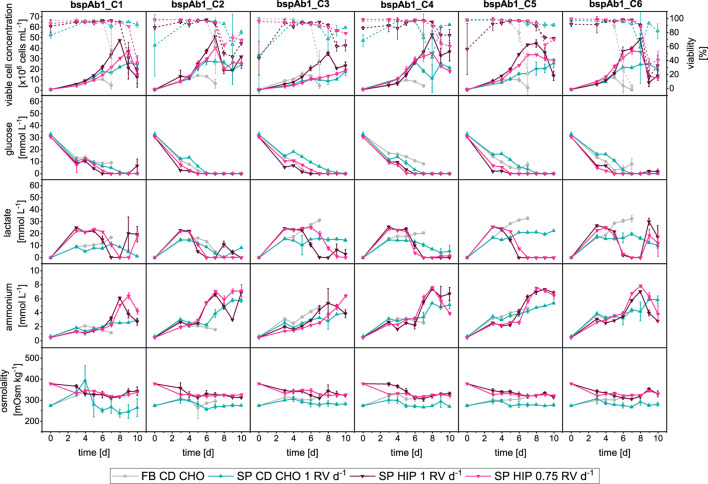
Overview of growth and metabolites for bspAb1 CHO cell clone screening in MWPs using fed-batch and semi-perfusion methodologies. Cells were inoculated at 1 × 10^6^ cells mL^−1^ for all used methodologies. For fed-batch cultures, cells were cultivated in CD CHO with a feed step from day 3 to day 7 (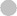). For semi-perfusion cultures, cells were cultivated in CD CHO (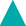) and HIP media (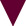) with a perfusion rate of 1 RV d^−1^ and in HIP medium with a perfusion rate of 0.75 RV d^−1^ (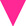). Row 1: viable cell concentration (closed, straight) and viability (open, dashed), Row 2: glucose concentration; Row 3: lactate concentration; Row 4: ammonium concentration; Row 5: osmolality. Columns display the 6 individual clones. Mean of N = 3 wells. Error bars indicate standard deviation.

Experiments comparing the different operation modes and media were repeated with 6 clones expressing a bispecific antibody (see Figure 6 and Supplementary Material).

The comparison of FB, SP-CD CHO and SP-HIP showed similar results regarding growth, and metabolism to those obtained for mAb1 (Figure 6). The most prominent difference was the much shorter duration of the FB cultures for the bspAb1 clones of just 7 days instead of 10 days, due to an earlier reduction of viabilities around day 5. Semi-perfusion cultures ran for 10 days, and although clones in SP-HIP obtained higher maximum VCCs than clones in SP-CD CHO, the latter had higher viabilities until the end of culture as seen with the mAb1 cell clones. Furthermore, the dynamics of the metabolite concentrations were similar for the mAb1 and bspAb1 cell clones. The glucose concentrations depleted earlier for the SP-HIP cultures around day 5, while for SP-CD CHO cultures glucose was depleted from around day 7. For lactate, only the SP-HIP cultured clones showed a reduction in concentration around days 5 and 6, which coincided with the depletion of glucose, while for clones in FB and SP-CD CHO lactate concentration remained stable throughout. Overall, lactate concentrations remained below 30 mmol L^−1^ and ammonium concentrations remained below 10 mmol L^−1^ with the sudden increase of concentration on day 5–6 for clones in SP-HIP. Moreover, the comparison of growth and metabolite dynamics for semi-perfusion with different perfusion rates (SP-HIP vs. SP-HIP-75%) were nearly identical, as seen for the mAb1 cell clones. The only exception was bspAb1_C3 where VCCs were much lower for SP-HIP-75% than for SP-HIP.

The ranking was performed as described before, first based on the q_P_ and second on the MI_CL_ strategy. In Supplementary Table S2 of the Supplementary Material, the q_P_ based clone rankings are presented, where a colour code was employed, assigning a colour to each clone ranging from dark blue (bspAb1_C1) to dark red (bspAb1_C8), aiming to simplify the visualisation of the ranking. In addition, the raw q_P_ values are reported in Supplementary Table S3, in range of 1.37–6.82, 11.07–23.90, 6.31–14.93 and 6.90–26.62 pg cell^-1^ d^-1^ for FB, SP-CD CHO, SP-HIP and SP-HIP-75%, respectively. The MI_CL_ was in range of 0.06–1.00, 0.39–1.00, 0.30–1.00 and 0.27–1.00 for FB, SP-CD CHO, SP-HIP and SP-HIP-75%, respectively.

While for mAb1, the same clone was ranked top for all operation modes (FB vs. SP-CD CHO vs. SP-HIP) when using the q_P_ strategy, the opposite was true for the bspAb1 cell line, where the worst clone was identical (bspAb1_C6; Supplementary Table S3). Similarly, some ranking differences between SP-CD CHO and SP-HIP were observed. For the bspAb1, the top clone differed while the following ranking positions were shared by the same clones, while for the mAb1 clones, the lower ranking positions differed, and the higher positions were the same. Nonetheless, general differences between operation modes (FB vs. SP) were found, for example, bspAb1_C2 was consistently ranked lower in SP (#5; q_P_: 14.2 pg cell^−1^ d^−1^ (11.07–23.90 pg cell^−1^ d^−1^) and 12.3 pg cell^−1^ d^−1^ (6.31–14.93 pg cell^−1^ d^−1^) for SP-CD CHO and SP-HIP, respectively) than in FB (#2; q_P_ 5.3 pg cell^−1^ d^−1^ (1.37–6.82 pg cell^−1^ d^−1^)) while bspAb1_C5 consistently ranked higher in SP (#3) than in FB (#5). For the comparison between partial and total medium exchanges different rankings were obtained where particularly higher ranks showed variation while the same two clones (bspAb1_C2 and bspAb1_C6) ranked at #5 and #6 for SP-HIP and SP-HIP-75% (Supplementary Table S3). The q_P_ for bspAb1_C2 was 12.34 and 11.22 pg cell^−1^ d^−1^, and for bspAb1_C6, q_P_ was 6.31 and 6.90 pg cell^−1^ d^−1^ for SP-HIP and SP-HIP-75%, respectively. It is worth noting that the q_P_ ranges achieved were larger for SP-CD CHO (11.07–23.9 pg cell^−1^ d^−1^) and SP-HIP-75% (6.90–26.62 pg cell^−1^ d^−1^) than for SP-HIP (6.31–14.93 pg cell^−1^ d^−1^), while for the mAb1 cell line all experiments in SP resulted in similar ranges of q_P_ (see Section 3.2.1; Supplementary Table S1).

**FIGURE 7:**
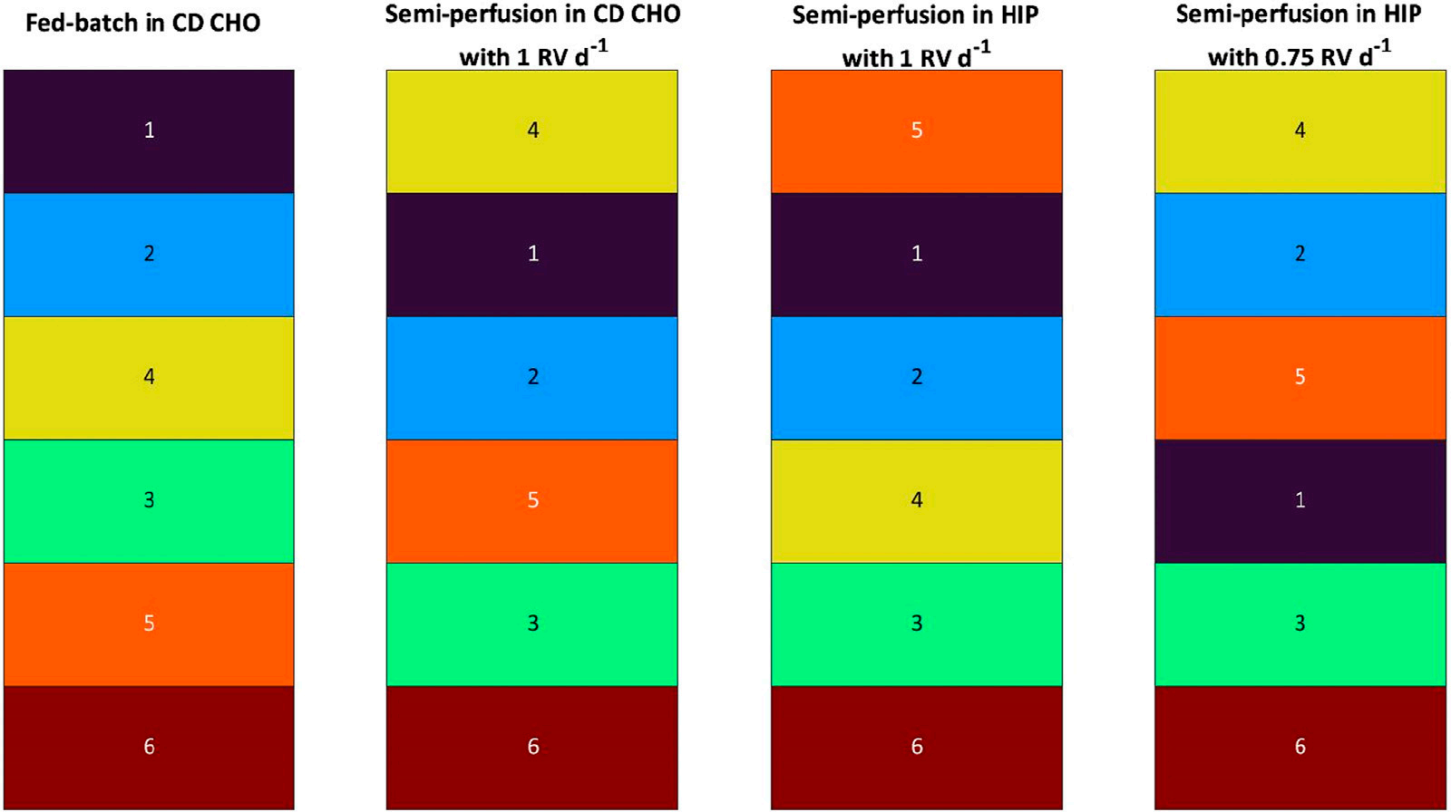
Ranking of 6 clones of bspAb1 CHO cell line based on Manufacturability index for fed-batch and semi-perfusion operation with total and partial medium exchanges in CD CHO and HIP medium.

When considering multiple parameters and using the MI_CL_ as basis for the ranking, FB and SP as well as SP with total and partial medium exchanges resulted in similarly different rankings as seen for q_P_ (Figure 7). However, the comparison across ranking strategies showed some interesting results, i.e., for both methods the FB ranking was identical. Further the worst performing clone was the same for all operation modes and perfusion rates and across ranking strategies (bspAb1_C6). However, for SP cultures some clones ranked consistently worse with the q_P_ strategy compared to the MI_CL_ strategy or vice versa. For example, bspAb1_C2 ranked at #5 and #3 for the q_P_ and MI_CL_ strategies, respectively, while bspAb1_C3 ranked at #5 for MI_CL_ and at the top two positions for the q_P_ strategy.

To assess applicability across cell lines and protein products, the study was replicated with a second cell line producing bspAb1. Similar to the mAb1 cell line, growth dynamics varied between FB and SP. While glucose, ammonium, and osmolarity concentrations exhibited comparable patterns across methods, lactate levels differed in SP-HIP and SP-HIP-75% cultures compared to FB and SP-CD CHO. Clone rankings revealed shared low-ranking clones, contrasting with the mAb1 cell line. Nevertheless, distinct rankings emerged between FB and SP with varying media and perfusion rates, corroborating previous findings.

## 4 Discussion

In this work, we examined eight cell clones producing mAb1 using fed-batch and semi-perfusion methods in a small volume platform. We assessed their growth and metabolic performance, and performed a cell clone ranking based on a single or a collection of parameters. This investigation was then repeated with six cell clones expressing bspAb1. To the best of our knowledge, this is the first comparison of cell clone rankings using both fed-batch and semi-perfusion methodologies. Our aim was to understand how the operation mode affects early-stage cell clone ranking.

### 4.1 Evaluation of growth and metabolic clone performance

In a first step, a screening of cell clones was carried out with a MWP methodology in fed-batch (Silk et al., 2010) and semi-perfusion at 1 RV d^−1^ (Tregidgo et al., 2023a) using fed-batch as well as perfusion specific media. The experiments aimed to understand what impact the media might have on the clone performance in addition to the shift in the operation mode.

For the mAb1 cell lines, the growth dynamics of SP cultures with total medium exchanges in both media were comparable and achieved a 2- to 5-fold increase in maximum VCCs compared to FB cultures. Similar patterns were observed for the bspAb1 cell clones (Figure 6). Further, viabilities of FB cultures decreased earlier than for SP cultures, which was most notable for the bspAb1 cell clones where the cultivation time of FB cultures was significantly shorter (7 days instead of 10 days).

The initial hypothesis was that glucose depletion, which was observed in all clones for all operation modes, in combination with the accumulation of toxic by-products such as lactate and ammonium, led to the reduced viability. Previous studies on CHO cells have shown a 25% reduction in growth at lactate concentrations of 60 mmol L^−1^ or ammonium concentrations greater than 10–15 mmol L^−1^ (Lao and Toth, 1997). However, lactate and ammonium concentrations of all clones in FB cultures (for both products) were shown to be similar or lower compared to SP cultures (in both media). Further, lactate and ammonium concentrations remained below 30 mmol L^−1^ and 10 mmol L^−1^, respectively (for all cultivations and cell lines) and thus below levels typically considered toxic. Moreover, in SP cultures a reduction of lactate concentration was observed, which coincided with the depletion of glucose and indicated a shift of metabolism from lactate production to consumption, as previously described in the literature for CHO cells (Altamirano et al., 2000; 2004; Mulukutla et al., 2012). Interestingly, only SP cultures in HIP medium (of both cell lines) showed a drastic reduction and depletion of lactate at the end of cultivation. The depletion of both glucose and lactate is the most likely cause for the reduced growth and greater reduction of viability in SP-HIP cultures compared to SP-CD CHO cultures, where lactate was not depleted.

In addition to the evaluation of operation modes, SP was investigated in greater detail by applying different perfusion rates. This aimed to understand potential differences of clone performance and resulting cell clone rankings between a typical manual SP operation with 1 RV d^−1^ whilst a prospective automated workflow would use a partial medium exchange (often at a fixed rate, e.g., not more than 75% of the working volume – 0.75 RV d^−1^) due to the operation with a liquid handling arm and the use of sedimentation as the cell retention method (Bielser et al., 2019; Jin et al., 2021). For both perfusion rates, the growth and metabolic performance was nearly identical for all the mAb1 and bspAb1 cell lines. A faster reduction and depletion of glucose in combination with an earlier change of metabolism to lactate consumption was expected for SP-HIP-75% compared to SP-HIP cultures, however the profiles obtained were very similar. The reason for this observation could be that a change in metabolism for the SP-HIP-75% cultured clones occurred, where higher lactate concentrations led to a reduction in lactate production rate and higher consumption rate even in the presence of glucose. Similar observations were previously made for batch and fed-batch CHO cultures (Altamirano et al., 2004; Dorai et al., 2009; Lao and Toth, 1997; Li et al., 2012).

### 4.2 Evaluation of the ranking

For the cell clone ranking, two strategies were used and compared. Previous studies conducted at mL-scale showed that clone ranking is often based on a single productivity parameter (Bielser et al., 2019; Markert et al., 2019). In batch and fed-batch operations the use of accumulated end-point titre is very common. However, in this work two distinctly different operation modes, FB and SP, were being compared. Thus, the selected parameter needed to account for the accumulation of the product in FB operation as well as the medium exchange leading to product removal in SP. The cell-specific production rate, q_P_, was suggested in the literature for early-stage screening at low cell concentrations to avoid early exclusion of clones (Markert et al., 2019). The calculation of q_P_ carried out in this work took the variable flow rates of SP and FB into account, allowing for a fair comparison between operation modes. In the first instance a clone ranking based on the q_P_ was carried out.

For mAb1, the ranking of clones for SP-CD CHO and SP-HIP was nearly identical with minor differences of low performing clones at positions #6 to #8. Thus, it was initially hypothesised that the medium had little impact on the outcome of the ranking. The comparison between operation modes (FB vs. SP-HIP) showed greater differences. Some clones ranked either high or low, while the top clone was identical for both operation modes. Some clones changed ranking positions quite significantly, and by more than 2 positions (e.g., mAb1_C6). Similar observations can be made regarding the comparison between the different perfusion rates (SP-HIP vs. SP-HIP-75%), where, for example, the top clone for SP-HIP-75% ranked at position #4 for SP-HIP. These results on the clone ranking based on the q_P_ led to the hypotheses that both (i) the operation mode and (ii) the perfusion rate impact the clone ranking. Consequently, the selection of clone candidates for transfer to perfusion bioreactors for further selective screening differs depending on the chosen method (operation mode) and the use of a manual or automated workflow (perfusion rate).

In a complementary study, the bspAb1 cell clone ranking between FB and SP showed more ranking position changes than observed for the mAb1 cell line ranking. For example, bspAb1_C2 performed considerably worse in SP culture (#5) than in FB culture (#2). It should be noted that the rankings comparing SP-CD CHO and SP-HIP (1 RV d^−1^) showed some differences in the better performing clones as opposed to the worst performing for mAb1. It is noteworthy that this observation could have been caused by the smaller number of clones investigated for bspAb1. When comparing the clone rankings for SP cultures with total and partial medium exchanges (1 RV d^−1^ vs. 0.75 RV d^−1^), ranking differences for top performing clones were similar to the ranking of the mAb1 clones. Even though the number of clones investigated for bspAb1 was smaller than for mAb1, a trend seems to emerge showing that the cell clone ranking was being impacted by the operation mode and perfusion rate. However, these results also led to a re-evaluation of the impact of medium on the cell clone ranking. The exact compositions of the commercially-available media (ThermoFisher) are unknown, but it is stated that the HIP medium has a higher native concentration of many components compared to a FB medium (e.g., CD CHO), which is intended to be paired with a feed for best performance (ThermoFisher Scientific, 2024). While further studies testing additional media with larger panels of clones would give more insight on the impact of media on ranking, the influence is likely to be minor in comparison to the impact the different operation modes and the perfusion rates have. Additionally, investigations under glucose access should be considered. A preliminary clone screening with additional glucose supplementation in the perfusion-specific medium (HIP) and total medium exchange was performed with the mAb1 clones (referred to as SP-HIP20; see Supplementary Figure S1; Supplementary Table S2). The investigation showed only minor differences in single-parameter ranking between conditions with or without glucose supplementation similar to differences between different media while significant differences remained evident between operation modes. Given these findings, we chose to proceed with glucose-limited conditions, as real-time glucose control is generally not feasible at this scale during cell clone screening. Medium optimisation is typically carried out in subsequent studies focused on the lead clone, conducted at bioreactor scale, where glucose concentrations will be measured, monitored, and controlled in real-time to maintain a predetermined range.

The small working volume of well plates in the initial cell clone screening often limits the number of studied parameters to VCCs and titre. However, the need for greater predictability and process robustness has driven the efforts to develop high-throughput analytical technologies using less volume per single analysis, thus allowing the analysis of additional parameters (e.g., metabolites). Although the process operation of well plate systems often differs from later stage bioreactor operations (i.e., orbital shaking vs. stirring), early insights in growth and metabolic behaviour as well as process performance can be valuable for process development and clone selection. Previous studies have shown that the MWP and a perfusion bioreactor compare well regarding the growth and production performance (Tregidgo et al., 2023a). We also performed comparability studies for 3 of the 8 clones, comparing MWP data (1 RV d^−1^) with historical data for 7 L perfusion bioreactors (1.2 RV d^−1^). These showed consistently good comparability for both growth and productivity metrics (data not shown).

The MWP methods used in this study allowed the collection of enough material to measure several parameters of growth (i.e., VCC, viability), productivity (i.e., titre), and metabolism. Based on these measurements, additional parameters such as cell specific rates were determined. Some studies have included growth parameters (e.g., VCC or growth rate) into their ranking (Gagliardi et al., 2019) by assigning a score to each parameter and determining ranks based on an overall score calculated with equally weighted parameters. However, this approach is often performed manually and is limited to a small number of clones and parameters to be feasible. Industrial cell clone screening processes assess several hundreds of candidates in MWPs; therefore, the manual assessment of each parameter, the assignment of scores and determination of ranks for all clones is an impractical and daunting task. To address this need, Goldrick et al. have developed a new index, the manufacturability index (MI_CL_), to summarize the cell clone performance by combining several parameters into a single numerical value that considers both the ranking within each parameter as well as the importance of each parameter through weighting (Goldrick et al., 2023).

In a second instance, the MI_CL_ approach was adapted to the investigation in MWPs comparing the different operation modes and another clone ranking based on the MI_CL_ was performed. The parameters, such as yields and cell-specific rates, were carefully selected considering the different feeding and medium exchange protocols that lead to accumulation or dilution of metabolites and product, to allow a fair comparison between FB and SP. Ten parameters were selected in total (see Section 2.4), where the lactate concentration was included as the only volumetric parameter impacted by the differences in feed and medium exchange protocols. This decision was made based on the dual nature of lactate as a toxic by-product potentially inhibiting cell growth but also serving as carbon-source through metabolism shift from lactate production to consumption (Luo et al., 2021; Mulukutla et al., 2012; Pereira et al., 2018).

The ranking obtained using the MI_CL_ strategy showed a similar overall outcome as the ranking based on the q_P_ with regards to different rankings between operation modes as well as between perfusion rates (for both panels of cell lines). The results emphasise that the chosen methods (FB vs. SP) at MWP scale impact the candidate selection for further screening in larger scales, leading to exclusion of clones more suitable for perfusion processes while including clones more suitable for fed-batch.

The comparison across ranking methods (q_P_ vs. MI_CL_) gave some interesting insight. For example, the ranking for FB using either the single parameter q_P_ or the MI_CL_ metric was identical for both cell line panels, which suggests that for FB cultures the q_P_ is a good enough indicator for early stage ranking at MWP scale where availability of data is limited. However, for SP the same cannot be said, as some greater differences between ranking methods (q_P_ vs. MI_CL_) could be observed. The most prominent example was mAb1_C7 in SP-CD CHO, which ranked low (#6) for q_P_ but on top position for MI_CL_ ranking. The most likely explanation for this finding was the better growth observed for mAb1_C7 in SP-CD CHO, where viabilities were high throughout and the highest maximum VCCs was achieved, compared to FB, SP-HIP, or SP-HIP-75%. Another possible reason is that the medium does have some impact on the ranking. This was supported by the ranking results for the bspAb1 cell clones, where some differences between SP-CD CHO and SP-HIP were already observed for the ranking based on q_P_ and the ranking based on q_P_ and MI_CL_ for SP-HIP20 with higher glucose concentrations (see Supplementary Figures S1, S2; Supplementary Table S2). Another interesting example was clone mAb1_C6, which ranked overall better in SP cultures compared to FB, including SP-HIP-75% where it had ranked much lower for the q_P_ based ranking. This also led to an interesting observation comparing ranking methods (q_P_ vs. MI_CL_) for SP cultures. It seems that ranking results of SP cultures, regardless of the medium or the perfusion rate, are more consistent using the MI_CL_ strategy. With some exceptions, the same mAb1 clones cluster to rank at either higher or lower positions with mAb1_C1 being a “barrier” in the middle. For the bspAb1, the greater differences can likely be attributed to the smaller number of clones. Nonetheless, a clustering of clones at low-ranking positions can also be observed. This could be seen as supporting the hypothesis of a more consistent ranking across perfusion rates and media when using the MI_CL_ strategy for SP cultivations.

### 4.3 Evaluation of the reproducibility

Lastly, the reproducibility of the results was evaluated in this work. Particularly the semi-perfusion screening was of importance as the different ranking strategies showed the ranking positions of individual clones differed. To further investigate this, the SP-HIP-75% condition and the mAb1 clones were selected and experiments repeated to better understand the statistical variability.

The comparison of the two runs showed a close similarity of growth and metabolic performance, even though the second run showed slightly slower growth. A possible reason for this could be that Run 2 (P16) was initiated at a later passage number than Run 1 (P10). However, previous studies found no significant impairment of performance for passage numbers up to P20 (Kaur et al., 2023; Qian et al., 2020). Another likely cause for this observation might be the manual operation of this clone screening, which is more prone to operator error and day-to-day performance variation, thus potentially leading to less accurate medium exchanges or accidental disturbance of the cell pellet during the medium exchanges. It is expected that automating the workflow would lead to higher precision and less difference between identical runs, ultimately increasing reproducibility and robustness.

The clone ranking of both runs using the single parameter strategy based on the q_P_ is reported in Table 2 and showed that 4 out of 8 clones were ranked at identical positions including the two top clones. Of the remaining 4 clones, 2 showed changes by two ranking positions (from #3 to #5 and vice versa) while the remaining 2 showed even greater position changes. It is possible that the growth differences of Run 2 caused the observed variation in the q_P_ based ranking, though it is unlikely this is the only cause. For example, the clones mAb1_C2 and mAb1_C6 both achieved higher VCC in Run 1 than in Run 2, which would suggest that the productivity was higher in Run 1. However, while this assumption seems to be accurate for mAb1_C2, which ranked at position #3 in Run 1 and dropped by 2 ranking positions to #5 (Run 2), mAb1_C6 improved by 3 positions from #7 (Run 1) to #4 (Run 2).

For Run 1, the comparison of both ranking strategies (q_P_ vs. MI_CL_) had shown significant position changes for some clones (Section 3.2.2). Although the top two clones had been identical, some clones such as mAb1_C2 were ranked much lower while others (e.g., mAb1_C6) ranked higher with the MI_CL_ strategy. For Run 2 similar observations were made, where, for example, mAb1_C2 dropped from position #5 for q_P_ to #8 for MI_CL_. Comparing the rankings of the MI_CL_ strategy of both runs showed that only the top clone was identical in both runs. While the remaining positions did not share identical clones, a clustering of clones was observed, such that mAb1_C4, mAb1_C6 and mAb1_C8 ranked “high” in both runs, whereas the remaining clones ranked consistently “low”. In addition, the clones appeared in pairs, where two clones shared the same two positions in both runs (e.g., mAb1_1 and mAb1_C3 on positions #5 and #6). The small difference by one ranking position, can be attributed to the more error-prone manual operation of this investigation.

To summarize, although multiple clones ranked in the same position for the q_P_ strategy, as opposed to only one for the MI_CL_ strategy, the overall ranking based on q_P_ showed more variation than that based on MI_CL_. This was due to the fact that several clones changed their ranks by two or more positions for the q_P_ strategy, while for the MI_CL_ strategy, position changes were limited to only one rank (with the exception of mAb1_C8 which changed by two ranks). Consequently, a more consistent ranking pattern was obtained using the MI_CL_ strategy. This suggests that ranking based on MI_CL_ is generally more consistent, which is a hypothesis that was previously developed based on results from other SP conditions (Sections 3.2.2, 4.2).

## 5 Concluding remarks

The growing demand for biopharmaceuticals has created a need for more flexible manufacturing strategies. Particularly for the upstream bioreactor operation, efforts of academia and industry have focused on the process operational implementation and optimisation of perfusion bioreactors including the development of suitable scale-down models to mimic the continuous medium exchange. Yet, at the early stage of cell line selection, the fed-batch methodology remains the standard with the investigation and implementation of more representative methods for perfusion processes still wanting.

Our study highlights the importance of considering the operation mode during early cell clone screening. We used both fed-batch and semi-perfusion methods to screen 14 clones for two therapeutic protein products at MWP scale and evaluated their growth and metabolism followed by clone ranking utilising two different strategies based on either a single (q_P_) or a collection of several parameters (MI_CL_). We observed differences in productivity and growth performance between operation mode, which affected clone rankings. The ranking variations suggest that relying solely on traditional fed-batch methods for clone selection for perfusion processes might lead to overlooking clones better suited for perfusion. This highlights the importance of employing methods that mimic the intended production environment during cell clone selection at MWP scale.

Additionally, our findings highlight the advantages of incorporating multiple performance parameters into the evaluation process. The Manufacturability Index (MI_CL_) offered a more comprehensive assessment of clone performance compared to single-parameter approaches. While our study focused on an experimental framework, the integration of digital tools into cell clone screening could greatly enhance the selection process. Tools such as multivariate data analysis (MVDA) and machine learning algorithms are increasingly being utilized in bioprocessing, enabling real-time monitoring and in-depth analysis of cell performance. By incorporating these digital tools into cell clone screening analysis, we can streamline the process and facilitate more informed decision-making, ultimately improving the identification of promising clones early in development.

While our study focused on a limited number of clones due to manual operations, the consistent performance observed across multiple cell clones and products demonstrates the potential of our approach. Expanding the clone library and incorporating automation will enable more comprehensive and efficient validation of these findings. While product quality and genetic stability are typically not assessed at this early stage of clone screening, they become critical factors in the selection of the top 4–10 clones. However, advancements in analytical techniques may allow for the inclusion of these parameters in earlier developmental stages, further enhancing the screening and selection process. By leveraging the MI_CL_ ranking strategy and taking operational mode into account, we believe we can significantly accelerate biopharmaceutical development by identifying clones that are best suited for the desired production environment.

## Abbreviation

FB, Fed-batch; SP, Semi-perfusion; VCC, Viable cell concentration; q_P_, Cell-specific productivity; CHO, Chinese Hamster Ovary; MWP, Microwell plate; VCC, Viable cell concentration; mAb, Monoclonal antibody; bspAb, Bispecific antibody.

## Data Availability

The raw data supporting the conclusions of this article will be made available by the authors, without undue reservation.
