# Supplementary material for: Cell clone selection—impact of operation modes and medium exchange strategies on clone ranking
Source: Front Bioeng Biotechnol. 2025 Jan 20;12:1479633. doi: 10.3389/fbioe.2024.1479633 (PMC11788354; doi:10.3389/fbioe.2024.1479633)
Supplement: Supplementary file 1 [file DataSheet1.docx]

**Journal: Frontiers in Bioengineering and Biotechnology**

**Cell Clone Selection — Impact of Operation Modes and Medium Exchange Strategies on Clone Ranking**

**Marie Dorn^1^, Christine Ferng^3^, Kerensa Klottrup-Rees^2^, Kenneth Lee^3^, Martina Micheletti^1*^**

^1^Advanced Centre for Biochemical Engineering, Department of Biochemical Engineering, University College London, UK

^2^Cell Culture and Fermentation Sciences, Biopharmaceutical Development, AstraZeneca, Cambridge UK

^3^BioProcess Technologies and Engineering, Biopharmaceutical Developments, AstraZeneca, Gaithersburg, USA

*Corresponding author

Martina Micheletti

Advanced Centre for Biochemical Engineering, University College London, Bernard Katz Building, Gower Street, London, WC1E 6BT, UK

Email-address: m.micheletti@ucl.ac.uk

**Supplementary Material**

1. mAb1 cell line

Table S1: Summary of cell specific productivities and manufacturability index values of 8 mAb1 clones for fed-batch and semi-perfusion operations with total and partial medium exchanges.

| **Clones** | **q_P_ [pg cell^-1^ d^-1^]** | | | | | **MI_CL_** | | | | |
| --- | --- | --- | --- | --- | --- | --- | --- | --- | --- | --- |
|  | **FB** | **SP-CD CHO** | **SP-HIP** | **SP HIP-75%** | | **FB** | **SP-CD CHO** | **SP-HIP** | **SP HIP-75%** | |
|  |  |  |  | **Run 1** | **Run 2** |  |  |  | **Run 1** | **Run 2** |
| **mAb1_C1** | 6.08±0.89 | 28.86±3.10 | 29.69±4.46 | 26.98±4.41 | 18.99±5.25 | 0.48 | 0.94 | 0.58 | 0.69 | 0.57 |
| **mAb1_C2** | 7.38±1.20 | 24.03±2.86 | 23.47±4.18 | 27.58±5.53 | 23.66±2.09 | 0.54 | 0.69 | 0.57 | 0.63 | 0.43 |
| **mAb1_C3** | 5.04±0.83 | 18.41±2.87 | 17.55±2.42 | 20.18±7.62 | 18.81±1.78 | 0.47 | 0.82 | 0.52 | 0.68 | 0.65 |
| **mAb1_C4** | 25.47±4.85 | 41.95±4.09 | 39.14±3.48 | 32.14±7.44 | 35.13±2.15 | 1.00 | 0.97 | 0.78 | 0.89 | 0.82 |
| **mAb1_C5** | 9.58±1.82 | 24.87±5.59 | 29.45±9.35 | 46.57±13.98 | 40.98±3.73 | 0.92 | 0.95 | 1.00 | 1.00 | 1.00 |
| **mAb1_C6** | 5.40±1.71 | 30.08±3.27 | 33.06±3.37 | 22.13±8.04 | 28.77±1.18 | 0.47 | 0.96 | 0.80 | 0.75 | 0.746 |
| **mAb1_C7** | 1.64±0.39 | 19.64±1.42 | 16.46±2.27 | 16.46±2.29 | 12.21±2.15 | 0.14 | 1.00 | 0.43 | 0.43 | 0.54 |
| **mAb1_C8** | 9.55±2.52 | 17.31±1.11 | 16.58±2.56 | 24.94±3.37 | 24.94±3.37 | 0.55 | 0.62 | 0.38 | 0.69 | 0.86 |

Productivity values are given as average and standard deviation of N = 3 wells over 8 days from day 3 to day 10.

q_p_: cell specific productivity; MI_CL_: cell line manufacturability index

**mAb1 cell line – glucose supplementation investigation**

An additional cell clone screening was performed to investigate the impact of glucose depletion on the cell clone performance. Therefore, HIP medium was supplemented with 20% CHO CD Efficient Feed^TM^ B Liquid Nutrient Supplement (Gibco®, Thermo Fisher Scientific, Massachusetts, USA), in the following referred to as Feed B. According to the manufacturer, Feed B is a serum- and animal-free, chemically defined, glucose-rich liquid nutrient supplement. The experiment is referred to as SP-HIP20.


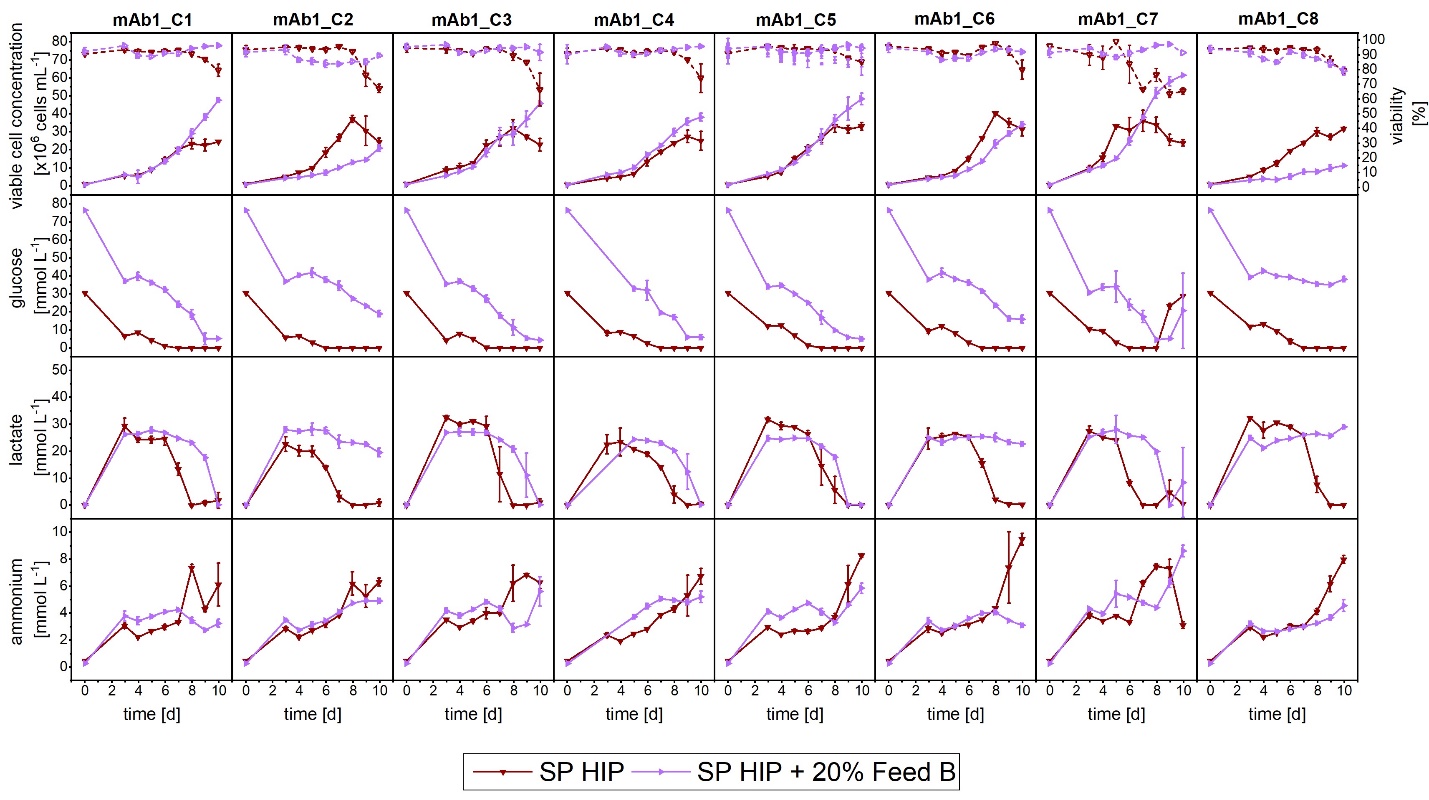


Figure S1: Overview of growth and metabolites for mAb1 CHO cell clone screening in MWPs using semi-perfusion methodologies. Cells were inoculated at 1 x 10^6^ cells mL^-1^ and cultivated in HIP medium (▼) and HIP medium supplemented with 20% Feed B (►). Row 1: viable cell concentration (closed, straight) and viability (open, dashed), Row 2: glucose concentration; Row 3: lactate concentration; Row 4: ammonium concentration. Columns display the eight individual clones. Mean of N = 3 wells. Error bars indicate standard deviation.

Table S 2: Ranking of eight mAb1 CHO cell clones based on average cell specific productivity values for semi-perfusion operation with total medium exchanges in HIP medium and HIP medium supplemented with 20% Feed B.

| **Ranking position** | **SP-HIP** | | **SP-HIP20** | |  |
| --- | --- | --- | --- | --- | --- |
|  | **1 RV d^-1^** | | **1 RV d^-1^** | |  |
| **#1** | mAb1_C4 |  | mAb1_C8 |  | |
| **#2** | mAb1_C6 |  | mAb1_C5 |  | |
| **#3** | mAb1_C1 |  | mAb1_C4 |  | |
| **#4** | mAb1_C5 |  | mAb1_C6 |  | |
| **#5** | mAb1_C2 |  | mAb1_C2 |  | |
| **#6** | mAb1_C3 |  | mAb1_C3 |  | |
| **#7** | mAb1_C8 |  | mAb1_C1 |  | |
| **#8** | mAb1_C7 |  | mAb1_C7 |  | |

Colour code to simplify the ranking visually.

Clones in SP-HIP20 conditions performed largely similar to clones of SP-HIP. The main differences identified were slightly higher growth for mAb1_C7, while mAb1_C8 showed significantly reduced growth. This reduced growth was evaluated as the main contributor to the top rank of mAb1_C8 in the SP-HIP20 condition due to the calculation of the q_P_. While some differences were observable these were evaluated as minor compared to the differences between operation mode. However, they do


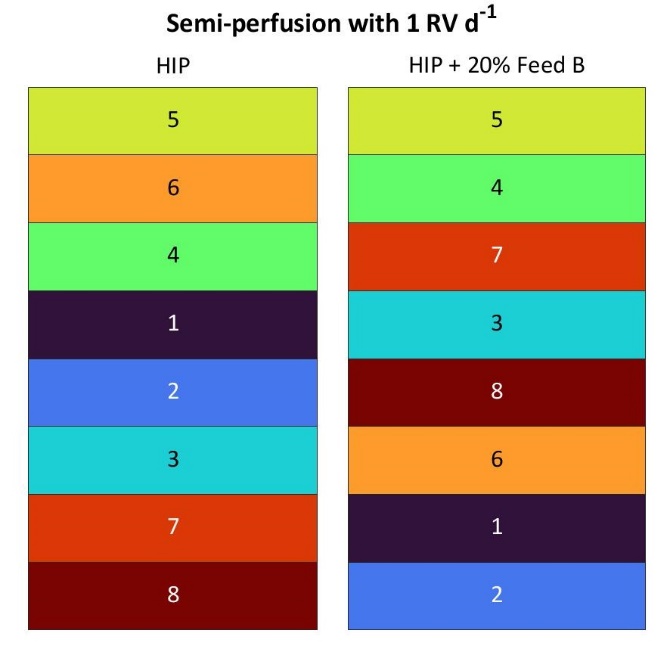


Figure S2: Ranking of eight mAb1 CHO cell clones based on manufacturability index for semi-perfusion operation with total medium exchanges in HIP medium and HIP medium + 20% Feed B.

1. bspAb cell line

Table S3: Ranking of six bspAb1 CHO cell clones based on average cell specific productivity values for fed-batch and semi-perfusion operation with total and partial medium exchanges in CD CHO and HIP medium.

| **Ranking position** | **FB** | | **SP-CD CHO** | | **SP-HIP** | | **SP-HIP-75%** | |
| --- | --- | --- | --- | --- | --- | --- | --- | --- |
|  | **-** | | **1 RV d^-1^** | | **1 RV d^-1^** | | **0.75 RV d^-1^** | |
| **#1** | bspAb_C1 |  | bspAb_C1 |  | bspAb_C4 |  | bspAb_C3 |  |
| **#2** | bspAb_C2 |  | bspAb_C3 |  | bspAb_C3 |  | bspAb_C1 |  |
| **#3** | bspAb_C4 |  | bspAb_C5 |  | bspAb_C5 |  | bspAb_C4 |  |
| **#4** | bspAb_C3 |  | bspAb_C4 |  | bspAb_C1 |  | bspAb_C5 |  |
| **#5** | bspAb_C5 |  | bspAb_C2 |  | bspAb_C2 |  | bspAb_C2 |  |
| **#6** | bspAb_C6 |  | bspAb_C6 |  | bspAb_C6 |  | bspAb_C6 |  |

Colour code to simplify the ranking visually. Average of q_P_ for FB cultures was calculated from day 3 to day 7, for SP cultures the average was calculated from day 3 to day 10.

Table S4: Summary of cell specific productivities and manufacturability index values of six bspAb1 CHO cell clones for fed-batch and semi-perfusion operations with total and partial medium exchanges.

| **Clones** | **q_P_ [pg cell^-1^ d^-1^]** | | | | **MI_CL_** | | | |
| --- | --- | --- | --- | --- | --- | --- | --- | --- |
|  | **FB** | **SP-CD CHO** | **SP-HIP** | **SP HIP-75%** | **FB** | **SP-CD CHO** | **SP-HIP** | **SP HIP-75%** |
| **bspAb1_C1** | 6.82±0.83 | 23.90±2.91 | 12.93±2.87 | 21.03±2.61 | 1.00 | 0.94 | 0.94 | 0.83 |
| **bspAb1_C2** | 5.28±0.69 | 14.20±1.93 | 12.34±1.83 | 11.22±2.05 | 0.81 | 0.83 | 0.90 | 0.98 |
| **bspAb1_C3** | 3.64±1.11 | 22.98±4.81 | 13.66±0.94 | 26.62±3.84 | 0.54 | 0.55 | 0.66 | 0.59 |
| **bspAb1_C4** | 4.80±2.51 | 14.28±4.19 | 14.93±2.03 | 20.26±1.94 | 0.54 | 1.00 | 0.84 | 1.00 |
| **bspAb1_C5** | 2.25±0.61 | 17.80±3.76 | 13.48±2.12 | 11.60±1.43 | 0.33 | 0.69 | 1.00 | 0.95 |
| **bspAb1_C6** | 1.37±1.23 | 11.07±0.35 | 6.31±1.08 | 6.90±0.36 | 0.06 | 0.39 | 0.30 | 0.27 |

Productivity values are given as average and standard deviation of N = 3 wells over 5 days for FB from day 3 to day 7 and 8 days from day 3 to day 10.

q_p_: cell specific productivity; MI_CL_: cell line manufacturability index
